# Endocytic function is critical for influenza A virus infection via DC-SIGN and L-SIGN

**DOI:** 10.1038/srep19428

**Published:** 2016-01-14

**Authors:** Leah Gillespie, Paula Roosendahl, Wy Ching Ng, Andrew G. Brooks, Patrick C. Reading, Sarah L. Londrigan

**Affiliations:** 1 Department of Microbiology and Immunology, University of Melbourne, at The Peter Doherty Institute for Infection and Immunity, Melbourne, Victoria 3000, Australia; 2 WHO Collaborating Centre for Reference and Research on Influenza, Victorian Infectious Diseases Reference Laboratory, at The Peter Doherty Institute for Infection and Immunity, 792 Elizabeth St, Victoria 3000, Australia

## Abstract

The ubiquitous presence of cell-surface sialic acid (SIA) has complicated efforts to identify specific transmembrane glycoproteins that function as *bone fide* entry receptors for influenza A virus (IAV) infection. The C-type lectin receptors (CLRs) DC-SIGN (CD209) and L-SIGN (CD209L) enhance IAV infection however it is not known if they act as attachment factors, passing virions to other unknown receptors for virus entry, or as authentic entry receptors for CLR-mediated virus uptake and infection. Sialic acid-deficient Lec2 Chinese Hamster Ovary (CHO) cell lines were resistant to IAV infection whereas expression of DC-SIGN/L-SIGN restored susceptibility of Lec2 cells to pH- and dynamin-dependent infection. Moreover, Lec2 cells expressing endocytosis-defective DC-SIGN/L-SIGN retained capacity to bind IAV but showed reduced susceptibility to infection. These studies confirm that DC-SIGN and L-SIGN are authentic endocytic receptors for IAV entry and infection.

Influenza A virus (IAV) can infect host cells via pH-dependent endocytosis. It is generally accepted that hemagglutinin (HA)-mediated recognition of cell surface sialic acid (SIA) is the first step in initiating IAV infection, however surprisingly little is known regarding the identity of specific receptors and/or coreceptors that mediate virus internalization. SIA structures do not exhibit signalling capacity but may act as attachment factors, promoting interactions with specific transmembrane receptors for virus uptake. Alternatively, recognition of critical SIA residues expressed by transmembrane receptors may be required to initiate virus entry. As SIA-independent entry and infection has also been reported^1^,^2^, it is possible that some receptors can bind IAV and signal independently of SIA, although attachment to SIA may concentrate virions at the cell surface and augment this mode of entry. The sorting of IAV into particular entry pathways will be determined by specific adaptor protein(s) that bind to the cytoplasmic tails of IAV receptors and co-receptors, activating intracellular signalling proteins for subsequent internalization of virus.

While little is known regarding the specific entry receptors for IAV expressed by epithelial cells, significant progress has been made towards identifying receptors that play a role in infectious entry of IAV into macrophages (MΦ) and dendritic cells (DC). C-type lectin receptors (CLRs) are transmembrane glycoproteins that recognize glycans expressed on IAV glycoproteins and a number of distinct CLRs have been implicated in promoting IAV infection (reviewed in^3^). Previous studies from our group implicated the macrophage mannose receptor (MMR) and the macrophage galactose-type lectin (MGL)-1 in infectious entry of IAV into mouse macrophages (MΦ)^4^,^5^,^6^. Of the human CLRs, expression of DC-SIGN (DC209) and/or L-SIGN (DC-SIGNR and CD209L) by transfected cell lines^7^,^8^,^9^ or primary cells^7^,^9^ has also been associated with enhanced susceptibility to IAV infection.

DC-SIGN and L-SIGN are tetrameric type II transmembrane CLRs expressing Ca^2+^-dependent (C-type) carbohydrate recognition domains (CRD) which bind preferentially to mannose-rich oligosaccharides (reviewed in^10^). Despite their similarities, DC-SIGN and L-SIGN differ with respect to tissue distribution. DC-SIGN is expressed by MΦ and DC subsets throughout the body whereas L-SIGN tends to be expressed by non-immune cells. In the respiratory tract, DC-SIGN is expressed by human alveolar MΦ and subpopulations of lung DCs^11^,^12^, whereas L-SIGN is expressed by bronchiolar epithelial cells, type II alveolar cells and endothelial cells of the lung^13^. A growing body of literature indicates that DC-SIGN and/or L-SIGN recognize glycans expressed by a range of different viruses to promote attachment and infection, as well as the capture and sequestration of virus, which may then be passed on to other permissive cells (reviewed in^10^).

The relevance of DC-SIGN in enhancing the infectious entry of IAV into primary human cells has been established in studies by Wang *et al*.^9^ and Hilliare *et al*.^7^, with both reporting that infection of monocyte-derived DCs could be blocked by anti-DC-SIGN mAb. In a previous study we demonstrated that SIA-deficient Lec2 CHO cells were resistant to IAV infection, however expression of DC-SIGN or L-SIGN by Lec2 cells resulted in Ca^2+^-dependent IAV attachment and enhanced susceptibility to IAV infection^8^. DC-SIGN/L-SIGN recognize glycans expressed on the viral HA and the neuraminidase (NA) glycoproteins. While the degree of HA glycosylation is highly variable between strains, levels of NA glycosylation tend to be more conserved (reviewed in^14^). Moreover, previous studies have demonstrated that the degree of glycosylation on the viral HA, rather than the NA, was critical in determining the ability of different viruses to infect Lec2 cells expressing either DC-SIGN or L-SIGN^8^. While we have some insight regarding the nature of IAV ligands recognized by CLR, the mechanisms by which DC-SIGN and L-SIGN promote IAV infection have not been defined. For example, DC-SIGN/L-SIGN may act as attachment factors, concentrating virions at the cell surface to promote interaction with other (unknown) receptors for infectious entry. Alternatively, recognition of IAV by DC-SIGN/L-SIGN may trigger direct receptor-mediated internalization, demonstrating their ability to act as *bone fide* entry receptors for IAV infection. The aim of this current study was to define the mechanisms by which DC-SIGN/L-SIGN act to enhance IAV infection.

Mutation of putative internalization domains in the N-terminal cytoplasmic tail of DC-SIGN (LL, YXXL and EEE) or L-SIGN (LL) has demonstrated the importance of the LL motif for efficient CLR-mediated endocytosis and trafficking^15^,^16^. Moreover, deletion of the entire cytoplasmic domain has been used to generate endocytosis-defective mutants of DC-SIGN/L-SIGN^16^,^17^. Herein, we demonstrate that Lec2 cells expressing DC-SIGN or L-SIGN with mutations (in which the dileucine motif (LL) was replaced by dialanine (AA)) or deletions (33 and 41 amino acids for DC-SIGN and L-SIGN, respectively) within the cytoplasmic tail bound IAV efficiently, but showed major defects in their ability to internalize monoclonal antibody (mAb) and virus, and in their susceptibility to IAV infection. Our studies confirm that both DC-SIGN and L-SIGN function as authentic receptors for IAV uptake and infection.

## Results

### Infectious entry of IAV into SIA-deficient Lec2 cells expressing DC-SIGN/L-SIGN occurs independently of SIA and is pH- and dynamin-dependent

**Figure 1:**
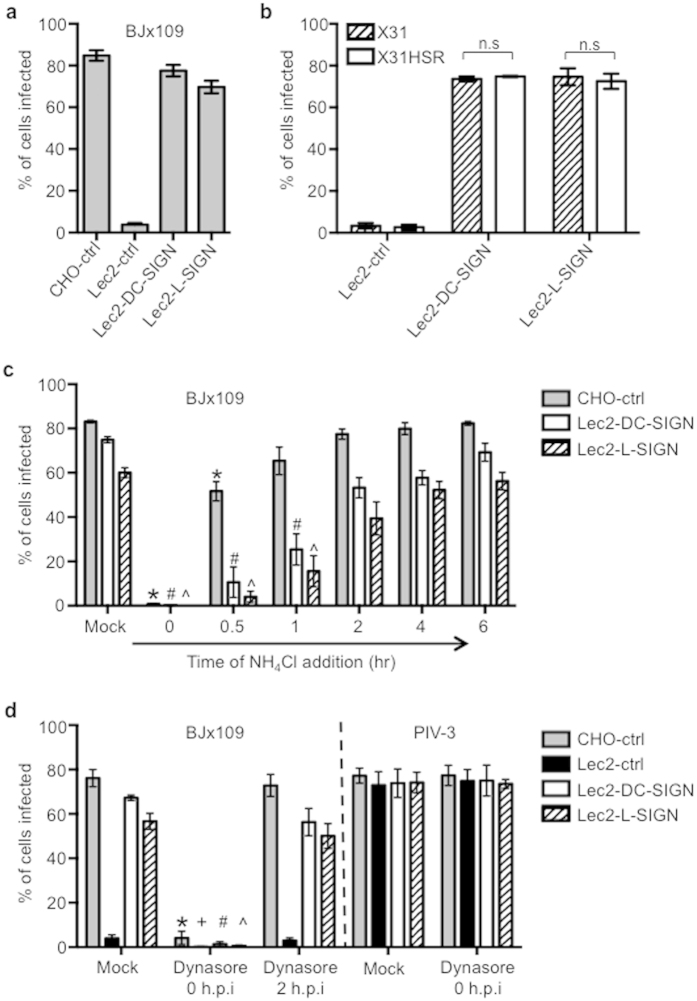
Infectious entry of IAV into SIA-deficient Lec2 cells expressing DC-SIGN/L-SIGN. (**a**,**b**) CHO-ctrl, Lec2-ctrl, Lec2-DC-SIGN or Lec2-L-SIGN cells were infected with (**a**) BJx109 or (**b**) HKx31 and HKx31 HS^R^ (MOI 50 PFU/cell) for 8 hr at 37 °C. Data represent the mean percent infected cells (±1 SEM) from at least 4 independent fields and are representative of multiple independent experiments. (n.s = no significant difference for HKx31 compared to HKx31 HS^R^ (Lec2-DC-SIGN; *P* = 0.37, Lec2-L-SIGN; *P* = 0.71) (Student’s *t*-test; two-tailed)). (**c**) CHO-ctrl, Lec2-DC-SIGN and Lec2-L-SIGN cells were incubated with BJx109 (MOI 50 PFU/cell) for 60 min at 37 °C and then washed and cultured 8 hr (mock). In some wells, 10 mM NH_4_Cl was added to the cells at the time of virus addition (t = 0 hr) or at various times thereafter to prevent further infection. All cells were incubated for 8 hr. Data represent the mean percent infected cells (±1 SEM). ^*#^^significantly different compared to mock for CHO-ctrl, Lec2-DC-SIGN and Lec2-L-SIGN cells, respectively. *p* < 0.05, one-way ANOVA followed by Tukey’s post-hoc test. (**d**) CHO-ctrl, Lec2-ctrl, Lec2-DC-SIGN and Lec2-L-SIGN cells were incubated with 10^7^ PFU of BJx109 (MOI 50 PFU/cell) for 60 min at 37 °C and then washed (mock). In some wells, 50 μM of dynasore was added to the cells at the time of infection (t = 0 hr) and replaced after washing, or dynasore was added two hours after incubation with virus (t = 2 hr). Cells were incubated for a total of 8 hr. Additional wells incubated with 10^6^ FFU of PIV-3 (MOI 5 FFU/cell) in the presence or absence of 50 μM dynasore were fixed and stained for expression of PIV-3 NP at 16 hr post-infection. Data represent the mean percent infected cells (±1 SEM). ^*+#^^significantly different compared to mock for CHO-ctrl, Lec2-ctrl, Lec2-DC-SIGN and Lec2-L-SIGN cells, respectively. *p* < 0.05, one-way ANOVA followed by Tukey’s post-hoc test. Data in (**c**,**d**) are pooled from 3 independent experiments.

Our previous studies have demonstrated that SIA-deficient Lec2 CHO (Lec2-ctrl) cells were resistant to infection by IAV, whereas Lec2 cells expressing human DC-SIGN or L-SIGN were highly susceptible^8^. These findings were confirmed using IAV strain BJx109 (Fig. 1a). Previous studies also demonstrated that IAV infection of Lec2 cells expressing DC-SIGN/L-SIGN was inhibited by mannan, but not by pre-treatment of cells with bacterial sialidase^8^, arguing that infection of Lec2 cells expressing CLR occurred independently of SIA. However, IAV strains differ markedly in their preference for particular SIA linkages and we have not formally addressed if this impacts on the ability of IAV to infect Lec2 cells via DC-SIGN/L-SIGN. IAV strain HKx31 shows HA preference for α2,6-linked SIA whereas a horse-serum resistant (HS^R^) mutant of HKx31 shows preference for α2,3-linked SIA^18^, although both viruses express similar numbers of glycans on the viral HA. Despite differences in SIA specificity, HKx31 and HKx31 HS^R^ infected Lec2-DC-SIGN and Lec2-L-SIGN cells to comparable levels (Fig. 1b), consistent with the notion that glycosylation of the viral HA, rather than its specificity for particular SIA linkages, is the most important determinant of CLR-mediated infection.

As expression of a single CLR (either DC-SIGN or L-SIGN) restores the susceptibility of Lec2 cells to IAV infection, we can probe this unique experimental system to determine the mechanisms by which a single receptor can promote IAV infection. It is well established that IAV infection is pH dependent and that the acidic pH of the endosomes induces an irreversible conformational change in the viral HA, promoting membrane fusion and release of the viral capsid into the cytoplasm (reviewed in^19^). Therefore, we used NH_4_Cl to examine the rate of infectious entry of IAV into CHO-ctrl, Lec2-DC-SIGN or Lec2-L-SIGN cells. In these experiments, cell monolayers were inoculated with BJx109 virus for 1 hr at 37 °C, washed and cultured for various times before the addition of 10 mM NH_4_Cl to retain virus in endosomes and prevent further infection. All wells were fixed at 8 hr post-inoculation and stained for expression of viral NP. As seen in Fig. 1c, inoculation of CHO-ctrl, Lec2-DC-SIGN and Lec2-L-SIGN with BJx109 (with no NH_4_Cl added = mock) resulted in 60–80% infection 8 hr post-exposure whereas addition of NH_4_Cl immediately after addition of virus (t = 0 hr) reduced infection levels to <1%. Infectious entry of BJx109 into CHO-ctrl cells occurred during the first 30 min at 37 °C and reached maximum levels (i.e. equivalent to mock) by 2 hr. In contrast, entry via DC-SIGN or L-SIGN was minimal during the first 30 min and increased progressively, with maximum levels detected by 4–6 hr.

To gain insight regarding the mechanisms underlying infectious entry of IAV via DC-SIGN/L-SIGN, we included dynasore, a small molecule inhibitor of clathrin- and caveolin-mediated endocytosis^20^ (Fig. 1d). Compared to mock (i.e. no dynasore), addition of 50 μM dynasore at the time of virus inoculation (i.e. t = 0 hr) reduced levels of IAV (BJx109) infection to <5%, confirming that entry into all cell types occurs via dynamin-dependent mechanisms. Addition of dynasore 2 hr after inoculation had only minor effects on levels of IAV infectivity, confirming that the inhibitory effects of dynasore are not mediated at post-entry steps in the virus life cycle. Further, the observation that dynasore did not inhibit the ability of PIV3 to infect CHO-ctrl cells (Fig. 1d), confirmed the specificity of dynasore-mediated inhibition of IAV infection since PIV-3 infects cells in a pH-independent manner via direct fusion with the plasma membrane (reviewed in^21^).

### Generation of Lec2 lines expressing endocytosis-defective mutants of DC-SIGN and L-SIGN

**Figure 2:**
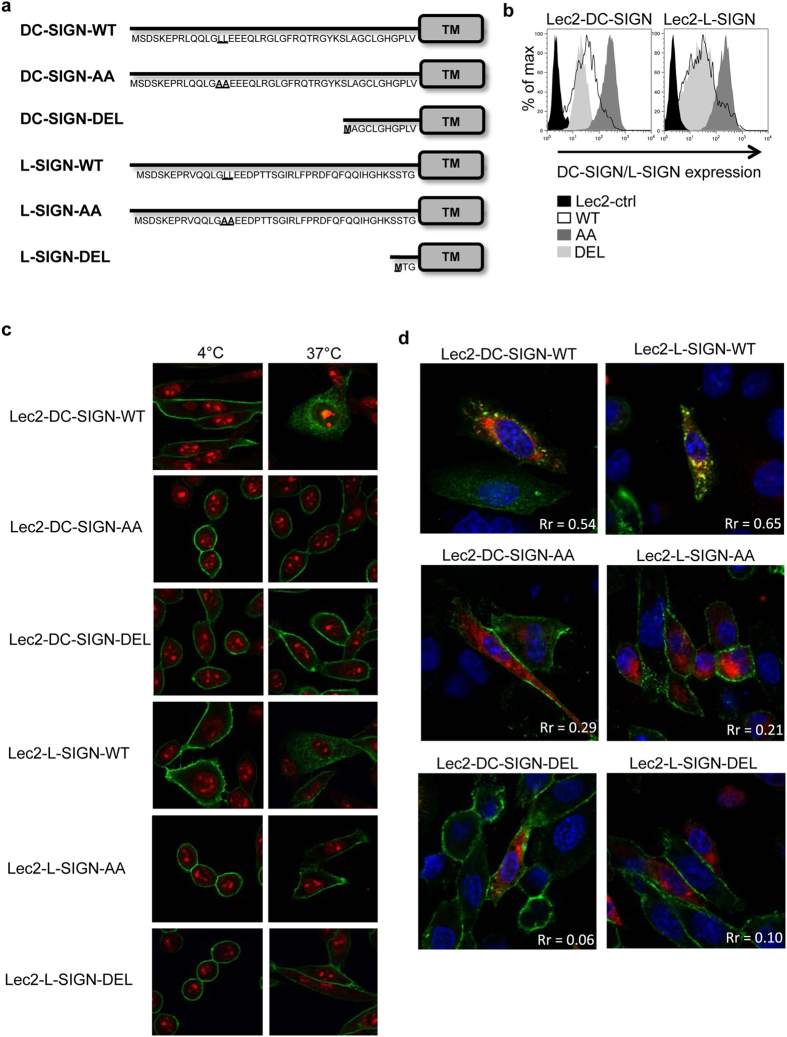
Generation and characterisation of cells expressing WT, AA or DEL mutants of DC-SIGN and L-SIGN. (**a**) Schematic overview of the CLR mutants. The amino acid sequence of the cytoplasmic domain of wild-type (WT) DC-SIGN and L-SIGN are shown, as well as mutants containing LL→AA mutations (AA) and deletion mutants lacking the entire cytoplasmic tail (DEL). Mutated residues are highlighted in bold and the transmembrane domain (TM) is shown. (**b**) FACS histograms showing cell-surface expression of DC-SIGN/L-SIGN on Lec2 cells expressing -WT, -AA or -DEL forms of the receptors. Lec2-ctrl cells were included for comparison. (**c**) Endocytic capacity of Lec2 cells expressing different forms of DC-SIGN/L-SIGN following cross-linking with antibody. Cell monolayers expressing -WT, -AA or -DEL forms of DC-SIGN/L-SIGN were incubated with anti-DC-SIGN/L-SIGN mAb for 1 hr at 4 °C, washed and incubated for a further 40 min either at 4 °C or 37 °C. Cells were then fixed and stained with Alexa Fluor-488 anti-mouse Ig (green, to detect anti-DC-SIGN/L-SIGN mAb) and propidium iodide (red, to stain the nucleus) and examined by confocal microscopy. (**d**) Cell monolayers were transiently transfected with a RFP-tagged Rab5 construct (red) and, 16 hr post-transfection, incubated with anti-DC-SIGN/L-SIGN mAb for 1 hr at 4 °C. After washing, cells were incubated at 4 °C or 37 °C for 40 min, and then fixed and stained with Alexa Fluor-488 anti-mouse Ig (green, to detect anti-DC-SIGN/L-SIGN mAb) and with DAPI (blue, to stain the nucleus). Co-localisation of Rab5 and DC-SIGN/L-SIGN was examined by confocal microscopy, where yellow staining is indicative of co-localisation.

Mutation of putative internalization domains (LL, YXXL and EEE for DC-SIGN, LL for L-SIGN) indicated that LL was the major motif contributing to the endocytic capacity of DC-SIGN and L-SIGN^15^,^16^. Therefore, we generated constructs expressing DC-SIGN/L-SIGN with the LL motif mutated to AA, or with the entire N-terminal intracellular domain of DC-SIGN (33 amino acids) or L-SIGN (41 amino acids) deleted (Fig. 2a). These constructs were used to generate stable Lec2 cell lines expressing wild-type (DC-SIGN-WT, L-SIGN-WT), mutated (DC-SIGN-AA, L-SIGN-AA) or deleted (DC-SIGN-DEL, L-SIGN-DEL) forms of each CLR. Cell surface expression of DC-SIGN and L-SIGN was confirmed using fluorescein-conjugated mAb 120612, which is cross-reactive between the two CLRs (Fig. 2b). Note that Lec2 lines expressing WT or DEL forms of DC-SIGN and L-SIGN showed similar levels of cell-surface receptor whereas Lec2-DC-SIGN-AA and Lec2-L-SIGN-AA lines expressed higher levels of cell-surface CLR.

To determine if mutation or deletion within the N-terminal intracellular domain abrogated the endocytic capacity of DC-SIGN/L-SIGN, cell monolayers were incubated with anti-DC-SIGN/L-SIGN mAb at 4 °C to facilitate binding to cell-surface CLR, then moved to 37 °C to promote internalisation. Following incubation at 4 °C, mAb bound to the surface of Lec2 cells expressing -WT, -AA and -DEL forms of DC-SIGN (Fig. 2c, upper panels) or L-SIGN (Fig. 2c, lower panels). When cell monolayers were moved to 37 °C, the anti-DC-SIGN/L-SIGN mAb redistributed into intracellular compartments in cells expressing WT DC-SIGN or L-SIGN (Fig. 2c), however the majority of mAb remained cell-surface associated for cells expressing -AA and -DEL mutants of either CLR. Note that mAb internalized by Lec2-DC-SIGN-WT or Lec2-L-SIGN-WT cells at 37 °C colocalized with Rab5^+^ compartments (Fig. 2d; Pearson’s coefficient: Rr = 0.54 and 0.65 respectively), whereas little colocalization was evident in Lec2 cells expressing -AA or -DEL mutants (Fig. 2d; Pearson’s coefficient: Rr < 0.5 for all mutants). Together, these data confirm that cross-linking of DC-SIGN and L-SIGN expressed by Lec2 cells results in endocytosis and delivery to Rab5^+^ early endosomes whereas mutation (LL to AA) within or deletion of the intracellular cytoplasmic domain abrogates endocytic capacity of DC-SIGN and L-SIGN.

### Lec2 cells expressing endocytosis-defective mutants of DC-SIGN/L-SIGN bind IAV efficiently, but exhibit defects in virus internalization and are resistant to IAV infection

**Figure 3:**
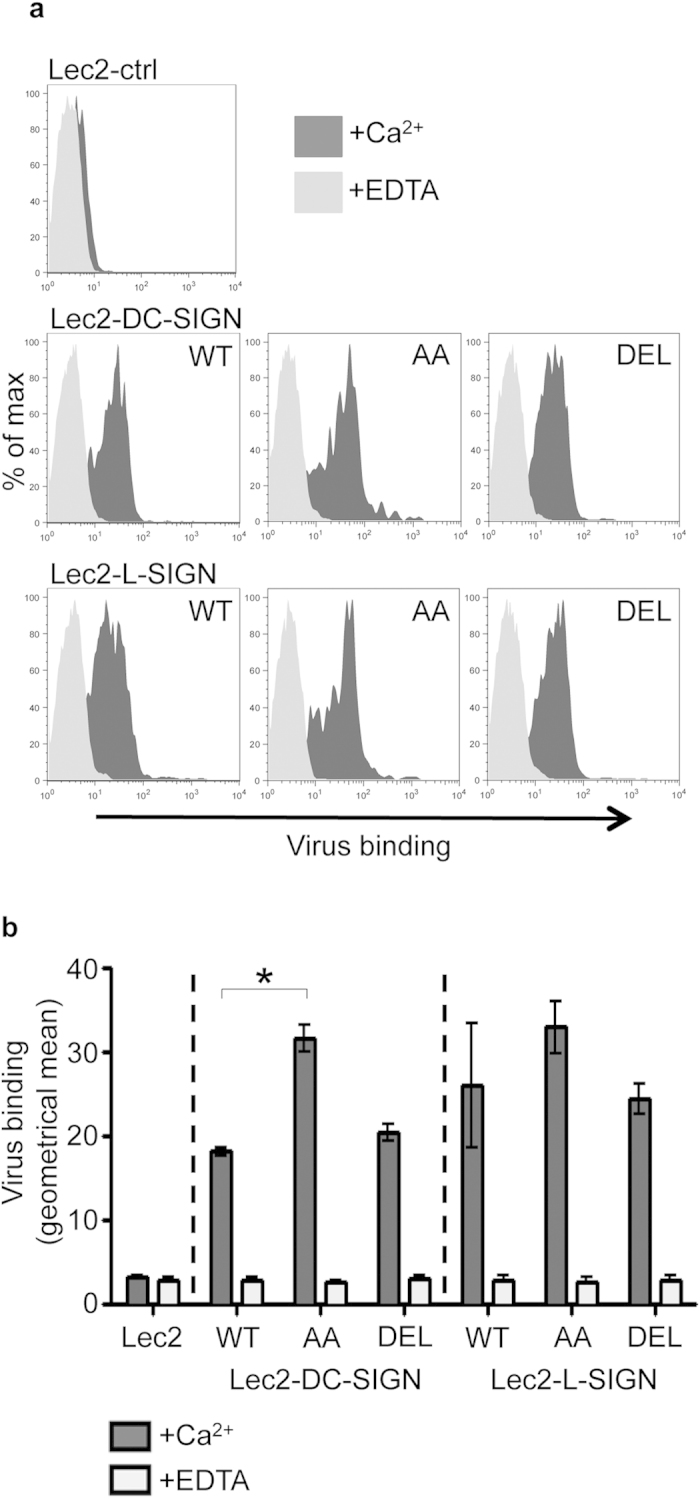
IAV binding to cells expressing WT, AA or DEL mutants of DC-SIGN/L-SIGN. (**a**) IAV binding to Lec2 cells expressing -WT, -AA or -DEL mutants of DC-SIGN/L-SIGN is Ca^2+^-dependent. Representative histograms show cell-surface binding of biotinylated IAV (strain BJx109) to Lec2 cells expressing -WT, -AA or -DEL forms of DC-SIGN/L-SIGN in the presence of 10 mM CaCl_2_ or 5 mM EDTA. No binding to Lec2-ctrl cells was observed. (**b**) IAV binding is presented as the geometrical mean of the fluorescence intensity (±1 SEM) from FACS histograms of triplicate samples. (*virus binding to Lec2-DC-SIGN-WT cells was significantly different compared with Lec2-DC-SIGN-AA cells. *p* < 0.05, One way ANOVA, followed by Tukey’s post-hoc test.)

We compared the ability of Lec2 cells expressing -WT, -AA and -DEL forms of DC-SIGN and L-SIGN to bind to IAV in the presence or absence of Ca^2+^. Representative histograms in Fig. 3a confirm that cell lines expressing WT and mutant forms of DC-SIGN/L-SIGN all bound IAV strain BJx109 in a Ca^2+^-dependent manner, consistent with CLR-mediated recognition of virus. Analysis of the geometrical means of the fluorescence intensity from triplicate samples indicated that -WT and -DEL mutants exhibited similar capacity to bind IAV whereas -AA mutants had a tendency to bind increased amounts of virus (Fig. 3b), consistent with enhanced cell-surface expression of -AA compared to -WT and -DEL on the Lec2 lines used in these studies (Fig. 2b).

**Figure 4:**
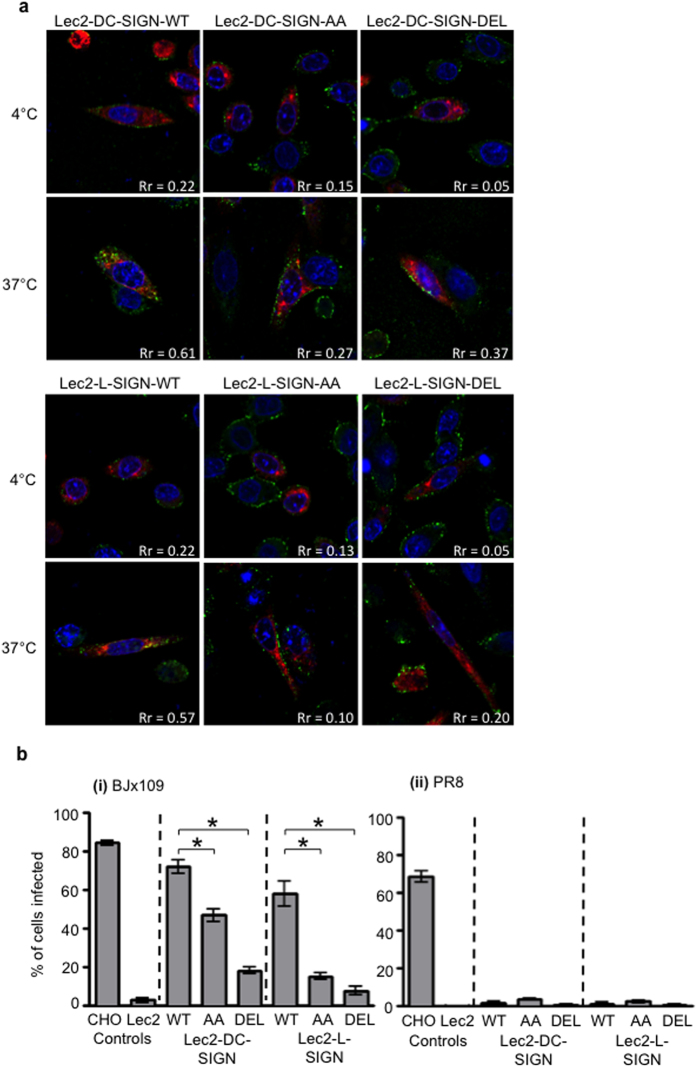
IAV entry into cells expressing WT, AA or DEL mutants of DC-SIGN/L-SIGN. (**a**) Monolayers of Lec2 cells expressing -WT, -AA or -DEL mutants of DC-SIGN/L-SIGN were transiently transfected with a RFP-tagged Rab5 construct (red) and, 16 hr post-transfection, incubated with 10^7^ PFU of BJx109 for 1 hr at 4 °C. After washing, cells were incubated at 4 °C or 37 °C for 40 min, and then fixed and stained with biotinylated anti-HA mAb, followed by streptavidin-488 (virus; green), and with DAPI to stain the nucleus (blue). Co-localisation of Rab5 and virus was examined by confocal microscopy (where yellow staining is indicative of co-localisation between virus and Rab5). (**b**) Monolayers of CHO-ctrl and Lec2-ctrl cells, as well as Lec2 cells expressing -WT, -AA and -DEL DC-SIGN/L-SIGN, were incubated with 10^7^ PFU (MOI 50 PFU/cell) of (i) BJx109, or (ii) PR8 for 60 min at 37 °C. After washing, cells were incubated a further 7 hr, then fixed and stained by immunofluorescence for expression of newly-synthesised viral NP. Data represent the mean percent infection (±1 SEM) from 3 independent experiments. *significantly different compared to Lec2-DC-SIGN-WT (for cells expressing AA or DEL forms of DC-SIGN) or Lec2-L-SIGN-WT (for cells expressing AA or DEL forms of L-SIGN). *p* < 0.05, One way ANOVA, followed by Tukey’s post-hoc test.

To assess IAV uptake by Lec2 cells expressing -WT, -AA and -DEL forms of DC-SIGN/L-SIGN, cell monolayers transfected with RFP-tagged Rab5 were incubated with BJx109 at 4 °C (to facilitate virus binding) and then moved to 37 °C (to facilitate virus uptake). At 4 °C, IAV was associated with the surface of Lec2 cells expressing various forms of DC-SIGN (Fig. 4a, upper panels) or L-SIGN (Fig. 4a, lower panels). After incubation at 37 °C, only cells expressing DC-SIGN-WT or L-SIGN-WT effectively internalized virus, as indicated by enhanced colocalization with Rab5^+^ compartments (Fig. 4a; Pearson’s coefficient: Rr = 0.61 and 0.57, respectively, compared to Rr values of <0.5).

Next, we compared the ability of BJx109 to infect Lec2 cell lines expressing WT and mutated forms of DC-SIGN/L-SIGN, as measured by immunofluorescent staining of viral NP at 8 hr post-infection. For Lec2 cells expressing DC-SIGN, the AA mutation was associated with a significant reduction in sensitivity to infection by BJx109 and infection was further reduced in Lec2-DC-SIGN-DEL cells (Fig. 4b(i)). For cells expressing L-SIGN, the AA mutation or deletion of the intracellular domain resulted in a profound reduction in susceptibility to BJx109 infection compared to cells expressing WT CLR (Fig. 4b(i)). The BJx109 strain expresses 4 potential sites of *N*-linked glycosylation on the head of the viral HA whereas the PR8 strain lacks glycosylation on the head of its HA glycoprotein^5^,^6^,^22^. Sialylated CHO-ctrl cells were susceptible to infection by PR8 whereas SIA-deficient Lec2-ctrl cells were not (Fig. 4b(ii)). Moreover, expression of -WT, -AA or -DEL forms of DC-SIGN or L-SIGN did not restore susceptibility to infection, confirming the critical role of HA glycosylation in determining sensitivity to CLR-mediated infection.

## Discussion

It is well established that DC-SIGN and L-SIGN are mannose-specific CLRs that act as attachment factors and promote infection by a range of different viruses, including Ebola and Marburg viruses, dengue virus (DV), human immunodeficiency virus (HIV), hepatitis C virus (HCV) and phleboviruses (reviewed in^3^,^23^). In most studies the mechanism/s underlying CLR-mediated infection enhancement have not been defined. However, it is clear that DC-SIGN can enhance infection of viruses by mechanisms that are dependent and independent of DC-SIGN-mediated endocytosis. For example, cells expressing WT or endocytosis-defective (LL and DEL) mutants of DC-SIGN showed similar sensitivity to DV infection^16^. Similarly, mutations within the cytoplasmic tails of DC-SIGN/L-SIGN that prevent efficient internalization of mAb-lectin complexes did not abrogate augmentation of Ebola virus infection^17^. Thus DC-SIGN and/or L-SIGN appear to act as attachment factors for these viruses, enhancing infection via cooperation with one or more additional cellular receptors. In contrast, cells expressing endocytosis-defective (LL) mutants of DC-SIGN were resistant to pH-dependent infection via arthropod-borne phleboviruses^24^, confirming that DC-SIGN can act as an authentic receptor for viral attachment and endocytosis, leading to infectious entry. Our findings that AA and DEL mutants of DC-SIGN/L-SIGN showed no defects in their ability to bind IAV but differed dramatically in their ability to facilitate endocytosis of mAb and virus, as well as their susceptibility to IAV infection, demonstrate that both DC-SIGN and L-SIGN represent attachment and internalization receptors for IAV. Hilliare *et al*.^7^ reported that desialylated DCs remained somewhat susceptible to IAV infection and that infection of desialylated DCs was further reduced in the presence of an anti-DC-SIGN mAb, indicating that CLR-mediated infection of primary cells can occur independently of cell surface SIA. We previously reported that pre-treatment of CHO cells with bacterial sialidase abrogated IAV infection but did not alter infection of Lec2 cells expressing either DC-SIGN or L-SIGN^8^. These studies confirm that high level expression of CLRs on SIA-deficient transfectants bypassed the requirement for SIA-mediated attachment. Herein, we confirm that DC-SIGN or L-SIGN expression on SIA-deficient Lec2 cells promotes CLR-mediated attachment, endocytosis and targeting of IAV to acidified endosomes, resulting in IAV infection, albeit at a somewhat slower rate than for sialylated CHO cells (Fig. 1c). Previous studies using epithelial cells have demonstrated that when one entry pathway is blocked (for example, clathrin- mediated, caveolin-mediated or macropinocytosis), IAV can enter or infect via another pathway, suggesting multiple routes of infectious entry into sialylated epithelial cells^25^,^26^,^27^. Therefore, sialylated parental CHO cells are likely to express multiple different attachment and entry receptors to facilitate infectious entry and this may be an important factor determining the more rapid infection of these cells compared to SIA-deficient CLR-expressing transfectants.

Identification and characterization of specific transmembrane glycoproteins that function as attachment factors and entry receptors for IAV has been complicated by the ubiquitous presence of SIA on the surface of mammalian cells. While interactions between IAV and epithelial cells induce signalling components implicated in virus uptake, such as phosphatidyl inositol 3-kinase (PI3K)^28^ and protein kinase C (PKC)^29^, it is not clear how virus binding transmits signals across the plasma membrane. Recently, multivalent binding of IAV to cell-surface SIA was reported to generate a signalling platform at the plasma membrane, activating receptor tyrosine kinases (RTKs) and subsequently intracellular signalling molecules such as PI3K^30^. We demonstrate that in the absence of SIA, IAV binding to DC-SIGN or L-SIGN can also activate cellular signalling pathways to induce dynamin-dependent internalisation of virus. Although the specific signalling components are yet to be elucidated, our data clearly implicate the LL motif in the cytoplasmic tail of DC-SIGN and L-SIGN in receptor-mediated IAV entry and infection.

Cross-linking of cell-surface DC-SIGN with mAb has been shown to induce efficient endocytosis of DC-SIGN in DCs^31^, suggesting that IAV-induced clustering of DC-SIGN/L-SIGN may activate signalling pathways that induce dynamin-dependent uptake of the receptor cluster and associated virus. However, while ligation with mAb relocalizes DC-SIGN to late endocytic and lysosomal compartments^15^, DC-SIGN is retained in early endosomes following uptake of HIV-1^32^, suggesting that cargo-dependent signalling may determine the particular internalization pathway utilized. DC-SIGN-mediated uptake of soluble antigens is impaired in the presence of clathrin inhibitors^33^ and LL-based motifs, like those in the cytosolic tail of DC-SIGN and L-SIGN, are involved in the uptake of various cargo into clathrin-coated vesicles^34^. L-SIGN lacks a number of the putative signalling domains expressed in the intracellular domain of DC-SIGN and much less is currently known regarding entry pathways utilized for L-SIGN-mediated endocytosis.

## Materials and Methods

### Cell lines

CHO Pro-5 cells were obtained from the American Type Culture Collection (ATCC), Manassas, VA, USA. The glycosylation mutant cell line, Lec2, derived from the CHO Pro-5 cells, was also obtained from the ATCC. Both cell lines were cultured in alpha-minimal essential medium (αMEM; Gibco-BRL, New York, USA) supplemented with 10% (vol/vol) fetal calf serum (FCS; JRH Biosciences, Kansas, USA), 4 mM L-glutamine, 100 IU penicillin, 10 μg of streptomycin/ml, non-essential amino acids (Gibco-BRL), 50 μM of β-mercaptoethanol and 1 mg of geneticin (G418)/ml (Gibco-BRL).

### Viruses

The IAV strains used in this study were BJx109 (H3N2, a high-yielding reassortant of A/PR/8/34 (PR8, H1N1) with A/Beijing/353/89 (H3N2) that expresses the H3N2 surface glycoproteins) and PR8 (A/PR/8/34, H1N1). HKx31 (also known as X-31, a reassortant of PR8 with A/Aichi/2/68 (H3N2), bearing the H3N2 surface glycoproteins) and horse-serum resistant HKx31 (HKx31 HS^R^)^18^ were also used. Viruses were grown in 10-day old embryonated eggs and titrated on Madin-Darby canine kidney (MDCK) cells by standard procedures and expressed as plaque forming units (PFU)/ml)^35^. Viruses were purified from allantoic fluid by rate zonal sedimentation on 25 to 75% (wt/vol) sucrose gradients, as described previously^35^. Purified virus was biotin labelled via exposed lysine groups using EZ-link-Sulfo-NHS-LC-LC Biotin reagent (Thermo Scientific, Illinois, USA) according to manufacturer’s instructions, dialysed against Tris-buffered saline (TBS, 0.05 M Tris-HCL, 0.15 M NaCl, pH 7.2) and stored at 4 °C. Parainfluenza virus type-3 (PIV-3) from the Victorian Infectious Diseases Reference Laboratory, Victoria, Australia was propagated in HEP-G2 cells. Titres of infectious virus were determined following immunofluorescence staining of HEP-G2 monolayers and expressed as fluorescent-focus units (FFU)/ml.

### Generation of Lec2 cells expressing DC-SIGN/L-SIGN or DC-SIGN/L-SIGN with mutations or deletions in the cytoplasmic tail

DC-SIGN and L-SIGN wild-type (WT) pcDNA3.1/V5-His-TOPO expression vectors have been described previously^8^. DC-SIGN/L-SIGN LL→AA were generated by site-directed mutagenesis (Quickchange kit, Invitrogen, Carlsbad, USA) and inserted into pcDNA3.1/V5-His-TOPO vectors. PCR-generated deletion (DEL) mutants of DC-SIGN or L-SIGN lacking the last 33 or 41 amino acids of the cytoplasmic tail, respectively, were also inserted into pcDNA3.1/V5-His-TOPO vectors. Nucleotide sequences of all DC-SIGN/L-SIGN constructs were confirmed by sequence analysis.

Lec2 cells were transfected with pcDNA3.1/V5-His-TOPO expression vectors containing WT, mutated (AA) or deleted (DEL) DC-SIGN/L-SIGN using FuGene 6 transfection reagent (Roche Diagnostic, Switzerland) according to manufacturer’s instructions. As controls, CHO and Lec2 cells were transfected with pcDNA3.1/V5-His-TOPO expressing cytoplasmic hen egg ovalbumin (OVA) lacking the sequence for cell-surface trafficking, as described^8^. Stable transfectants expressing wild type (Lec2-DC-SIGN-WT, Lec2-L-SIGN-WT), mutated (Lec2-DC-SIGN-AA, Lec2-L-SIGN-AA) or deleted (Lec2-DC-SIGN-DEL, Lec2-L-SIGN-DEL) forms of DC-SIGN/L-SIGN, or cytoplasmic OVA (CHO-ctrl, Lec2-ctrl) were selected in the presence of 1 mg/ml geneticin (G418; Invitrogen). Transfected cells were screened for cell-surface CLR expression using a fluorescein-conjugated MAb (clone 120612; R&D Systems, Minneapolis, USA) which is cross-reactive between DC-SIGN/L-SIGN and single cells with high cell-surface CLR expression were sorted using a FACSAria cell sorter (BD Biosciences, CA, USA) and expanded in culture for use in experiments.

### Binding of anti-DC-SIGN/L-SIGN mAb and IAV to cells

For analysis of mAb and IAV binding by flow cytometery, adherent cell lines were detached from plastic flasks using 0.75 mM EDTA in phosphate-buffered saline (PBS). After washing, cells were incubated with fluorescein-conjugated anti-DC-SIGN/L-SIGN mAb (clone 120612, R&D Systems) as per manufacturer’s instructions in FACs buffer (PBS supplemented with 1% FCS (vol/vol)) at 4 °C for 40 min. After incubation, cells were washed and levels of mAb binding determined using flow cytometry. For IAV binding, detached cells were washed and all subsequent steps performed in lectin buffer (TBS containing 10 mM CaCl_2_ and 1 mg of bovine serum albumin (BSA)/ml). Cells were incubated with 5 μg/ml of purified BJx109 in lectin buffer at 4 °C for 30 min. After washing, bound IAV was detected with 5 μg/ml biotinylated MAb C1/1 (a kind gift from Prof. Lorena Brown, Department of Microbiology and Immunology, University of Melbourne), followed by streptavidin-conjugated to allophycocyanin ((APC) Life Technologies, Eugene, USA) and flow cytometry. To determine if IAV binding to cells was Ca^2+^-dependent, CaCl_2_ was omitted from lectin buffer and replaced with 5 mM EDTA.

### Virus infection assays

Cells were seeded into 8-well chamber slides (Lab-Tek, Life Technologies) and infected with IAV as described previously^8^. Briefly, after overnight culture, slides with confluent cell monolayers were washed and incubated with IAV in serum-free αMEM for 1 hr at 37 °C (to allow virus binding and entry). After removal of virus inoculum cells were washed and incubated for a further 6–8 hr at 37 °C in serum-free αMEM. Slides were washed with PBS and then fixed with 80% (vol/vol) acetone. IAV-infected cells were stained using MAb MP3.10g2.1C7 (WHO Collaborating Centre for Reference and Research on Influenza, Melbourne, Australia), specific for the nucleoprotein (NP) of type A influenza viruses followed by FITC-conjugated goat anti-mouse Ig (Millipore, MA, USA). The percentage of infected cells was determined by co-staining with 4′,6-diamidoino-2-phenylindole (DAPI) and counting the total number of cells versus FITC positive cells under ×100 magnification. A minimum of four random fields were selected for counting, assessing at least 200 cells for each sample. In some experiments, cell monolayers were treated with 10 mM NH_4_Cl or 50 μM of dynasore (Sigma, St. Louis, USA) at various times as indicated.

For PIV-3, slides with confluent cell monolayers were washed and incubated with PIV-3 in serum-free αMEM for 1 hr at 37 °C. After removal of virus inoculum cells were washed and incubated for a further 16 hr at 37 °C in serum-free αMEM. Slides were washed with PBS and then fixed with 80% (vol/vol) acetone. PIV-3-infected cells were stained using a MAb specific for the HN protein (Abcam, Cambridge, UK) followed by FITC-conjugated goat anti-mouse Ig (Millipore).

### Internalisation of CLR-specific mAb or IAV

Cells seeded into 8-well chamber slides were cultured overnight, washed and incubated with anti-DC-SIGN/L-SIGN mAb (clone 120612) or with 1 × 10^8^ PFU/ml BJx109 (multiplicity of infection (MOI) 50 PFU/cell) in serum-free nutrient mixture F12K (Hams) media (Gibco-BRL) for 30 min at 4 °C (to allow mAb/IAV binding) or at 37 °C (to allow mAb/IAV entry). After this time, slides were washed with PBS and fixed in 80% (vol/vol) acetone. For binding and internalization of mAb, detection of mAb was enhanced using Alexa Fluor® 488 chicken anti-mouse Ig (Life Technologies, Eugene, USA). For binding and internalization of IAV, detection was performed using 5 μg/ml of biotinylated MAb C1/1, followed by Alexa Fluor® streptavidin-488 (Molecular Probes, Oregon, USA). All slides were co-stained with 4′,6-diamidino-2-phenylindole (DAPI) to detect cell nuclei and examined by confocal microscopy. Images were acquired with a Ziess LSM700 confocal microscope in conjunction with Zen2012 software. Rr values are indicative of colocalisation/correlation and were calculated using Image J software. In some experiments, cells were transfected with mRFP-Rab5 plasmid (#14437, Addgene, MA, USA) using Lipofectamine® 2000 transfection reagent (Life Technologies) according to manufacturer’s instructions. Cells were then cultured an additional 16 hours to allow for protein expression, washed and used in internalisation assays as described above.

### Statistical Analysis

Graphing and statistical analysis of data was performed using GraphPad Prism (GraphPad Software, San Diego, USA). An unpaired Student’s *t*-test was used to compare two sets of data. When comparing three or more sets of values, the data were analysed by one-way analysis of variance (ANOVA; nonparameteric), followed by post-hoc analysis using Tukey’s multiple comparison test. *p* values ≤ 0.05 were considered significant.

## Additional Information

**How to cite this article**: Gillespie, L. *et al*. Endocytic function is critical for influenza A virus infection via DC-SIGN and L-SIGN. *Sci. Rep.* **6**, 19428; doi: 10.1038/srep19428 (2016).
